# The role of SOX transcription factors in prostate cancer: Focusing on SOX2

**DOI:** 10.1016/j.gendis.2025.101692

**Published:** 2025-05-21

**Authors:** Guotu Du, Xiang Huang, Peng Su, Ying Yang, Shicheng Chen, Tianyu Huang, Neng Zhang

**Affiliations:** a Department of Urology, Affiliated Hospital of Zunyi Medical University, Zunyi, Guizhou 563003, China; b Department of Pathology, Affiliated Hospital of Zunyi Medical University, Zunyi, Guizhou 563003, China; c Department of Urology, The Second Affiliated Hospital of Zunyi Medical University, Zunyi, Guizhou 563000, China; d Department of Nursing, Affiliated Hospital of Zunyi Medical University, Zunyi, Guizhou 563003, China

**Keywords:** Lineage plasticity, Non-coding RNAs, Prostate cancer, SOX transcription factors, SOX2

## Abstract

Prostate cancer remains a major health problem, with its incidence ranking second among male malignancies worldwide. Recent studies have highlighted the critical role of the SOX family transcription factors, especially SOX2, in prostate cancer pathogenesis. SOX2 regulates the fate of cancer stem/progenitor cells, contributing to tumor initiation, development, and metastasis. Elevated SOX2 levels have been detected in prostate cancer tissues and are associated with higher tumor grade, aggressive phenotype, and poor prognosis. SOX2 also impacts various tumor biological behaviors, including cell proliferation, invasion, metastasis, resistance to apoptosis, and treatment resistance. This review highlights the role of SOX proteins in prostate cancer, focusing on the molecular mechanisms by which SOX2 drives cancer progression, elucidating the mechanisms controlling its activity, and emphasizing its potential as a therapeutic target.

## Introduction

Prostate cancer (PCa) is one of the most common malignancies among men worldwide. According to the 2022 GLOBOCAN statistics, PCa ranks second in incidence among male malignancies, only behind lung cancer.^1^ While the five-year survival rate for localized PCa approaches 100%, the survival rate drops significantly to 31% once diagnosed at an advanced stage.^2^ This dramatic decline in survival highlights the severity of disease progression, especially during the metastatic castration-resistant prostate cancer (mCRPC) phase, where tumors may transform into an androgen receptor (AR)-negative or neuroendocrine (NE) phenotype, complicating treatment and promoting resistance. As our understanding of the molecular mechanisms underlying PCa deepens, molecular subtyping has become an increasingly important tool for diagnosis and treatment. Using PAM50 testing, PCa is classified into three subtypes: luminal A, luminal B, and basal.^3^ In the castration-resistant prostate cancer (CRPC) stage, further classification based on the expression of AR- and NE-related genes allows for the identification of three distinct subtypes: AR^+^/NE^−^ CRPC, AR^−^/NE^−^ CRPC, and AR^−^/NE ^+^ CRPC.^4^ These subtypes offer new opportunities for personalized treatment; however, the management of CRPC remains a substantial challenge, particularly as tumors evolve into more aggressive forms, complicating therapeutic approaches.

In recent years, transcription factors of the SOX family have garnered significant attention from researchers. As the second member of the SOX family, SOX2 is one of the most extensively studied members and plays a crucial role in various cancer types. Specifically, SOX2 is involved in regulating tumor stem cell properties, the cell cycle, proliferation, metastasis, and treatment resistance. Although numerous reviews have addressed the role of the SOX family in cancers such as ovarian,^5^ cervical,^6^ and gastric cancer,^7^ a notable gap remains in the literature concerning the role of SOX2 in PCa. Thus, investigating the mechanisms through which SOX2 influences PCa is particularly important.

This review will focus on exploring the mechanisms of SOX2 in PCa, examining its potential roles in cancer initiation, progression, metastasis, and resistance. It will also discuss the prospects of targeting SOX2 as a therapeutic strategy. By integrating current research findings, this study aims to provide a theoretical foundation for the clinical application of SOX2 in PCa and offer insights into the development of novel treatment strategies for the future.

### SOX factors: an overview

In 1990, Sinclair et al^8^ and Gubbay et al^9^ cloned and confirmed the sex-determining region Y (*Sry*) box-containing gene in humans and mice. This discovery led to the identification of a series of homologous genes collectively termed the *SOX* gene family, which encode SOX proteins. A defining feature of *SOX* genes is their highly conserved high-mobility group (HMG) box, a DNA-binding domain consisting of 79 amino acids.^9^ By binding to target genes, SOX proteins play critical roles in processes such as sex determination,^10^ neural development,^11^^,^^12^ and cartilage differentiation.^13^ Aberrant expression of these proteins has been linked to tumorigenesis, influencing key tumor phenotypes, such as cell proliferation, invasion, migration, and resistance to treatment.^14^, ^15^, ^16^, ^17^

Transcription factors with amino acid sequences showing at least 50% homology to the HMG domain of SRY are classified as members of the SOX protein family.^5^ To date, more than 20 SOX transcription factors have been identified in humans and mice. Based on sequence similarity and domain structure, the SOX transcription factor family is subdivided into eight groups, designated SOXA through SOXH.^13^^,^^18^^,^^19^ Table 1 shows the SOX family members related to PCa.

**Table 1:** Overview of SOX family members related to human prostate cancer.

SOX family	SOX member	Expression concerning prostate cancer	Major outcomes	Chromosomal location	Reference
SOXA	SRY gene	–	–	Yp11.2	–
SOXB1	SOX1	Oncogenic	Regulates tumor invasiveness and tumor stem cell properties, and is up-regulated after androgen deprivation therapy	13q34	20,21
	SOX2	Oncogenic	Enhances proliferation, invasion, metastasis, prostatic ball formation, and cell cycle progression, inhibits apoptosis, and promotes tumor lineage plasticity and treatment resistance	3q26.33	22, 23, 24
	SOX3	–	–	Xq27.1	–
SOXB2	SOX14	Oncogenic	Signature gene related to metastasis	3q22.3	25
	SOX21	–	–	13q32.1	–
SOXC	SOX4	Oncogenic	Enhances epithelial–mesenchymal transition and tumor progression and metastasis, and promotes tumor pedigree plasticity and radio resistance	6p22.3	26, 27, 28, 29
	SOX11	Not determined	Some studies suggest that SOX11 plays a tumor-promoting role, promoting neuroendocrine differentiation, proliferation, and migration. However, other reports indicate that SOX11 acts as a tumor suppressor and is down-regulated in prostate cancer.	2p25.2	30, 31, 32
	SOX12	Oncogenic	–	20p13	–
SOXD	SOX5	Oncogenic	Initiates epithelial–mesenchymal transition	12p12.1	33,34
	SOX6	Tumor-suppressive	Inhibits cell proliferation	11p15.2	35
	SOX13	–	–	1q32.1	–
SOXE	SOX8	Oncogenic	Promotes the malignant biological behavior of enzalutamide-resistant LNCaP cells	16p13.3	36,37
	SOX9	Oncogenic	Promotes tumor cell proliferation and increases invasiveness and neural features	17q24.3	38,39
	SOX10	Tumor-suppressive	Controls invasion	22q13.1	40
SOXF	SOX7	Tumor-suppressive	Induces cell cycle arrest	8p23.1	40,41
	SOX17	Oncogenic	Regulates the sensitivity of enzalutamide-resistant cells to enzalutamide by modulating androgen receptor activity	8q11.23	42
	SOX18	Oncogenic	Enhances the proliferation, migration, and invasion abilities of prostate cancer cells *in vitro* and tumor growth *in vivo*	20q13.33	43
SOXG	SOX15	Tumor-suppressive	Positively regulates amine oxidase copper-containing 1 (AOC1) to inhibit tumor progression	17p13.1	44
SOXH	SOX30	Tumor-suppressive	Represses cell proliferation and invasion	5q33.3	45

### SOX2 expression and prognosis

SOX2, as a critical transcription factor, is closely linked to poor prognosis in PCa. In normal and benign prostatic hyperplasia tissues, SOX2 is predominantly expressed in basal cells. However, in high-grade prostatic intraepithelial neoplasia, SOX2 shows mixed staining in both basal and luminal cells.^23^ Studies have found that *SOX2* mRNA levels in PCa tissues are significantly higher than those in adjacent normal tissues, with even higher expression levels in recurrent PCa samples.^46^ Moreover, patients with higher *SOX2* expression levels have shorter biochemical recurrence-free survival, suggesting that SOX2 may play a role in PCa recurrence.^46^ However, other studies have reported no significant differences in *SOX2* gene expression between benign prostatic hyperplasia and PCa samples.^47^^,^^48^ At the protein level, immunohistochemical analysis with SOX2-specific antibodies has shown high expression of SOX2 in PCa tissues, and its expression intensity correlates positively with histological grade and Gleason score.^49^ Aboushousha and colleagues^50^ further validated this, showing that the proportion of SOX2-positive cells significantly correlates with Gleason grade, Gleason score, and grade group. They also observed that the proportion of SOX2-positive cells in PCa lesions was noticeably higher than in prostatitis or benign prostatic hyperplasia tissues.^50^ This finding contrasts with earlier studies, which suggested that the proportion of SOX2-positive cells decreases progressively in benign prostatic hyperplasia, high-grade prostatic intraepithelial neoplasia, and PCa.^51^ Further studies have shown that in primary prostate adenocarcinoma specimens, SOX2 is typically expressed negatively. However, SOX2 expression is significantly up-regulated in NE tumors and some mCRPC patients.^52^ Similar results have been observed at both the mRNA and protein levels, indicating that SOX2 is down-regulated in most PCa epithelial cells, with exceptions in a few low-grade and some high-grade PCa lesions. In these high-grade lesions, SOX2 protein is typically located in cancer cell clusters at the expanding or invasive front, where these cells are predominantly chromogranin-A-positive.^22^ Furthermore, research has shown that patients who experience biochemical recurrence tend to have higher median levels of *SOX2* mRNA, and those with higher *SOX2* expression tend to have shorter recurrence times. SOX2 has also been proposed as a potential functional biomarker for lymph node metastasis.^22^ In line with these findings, SOX2 is significantly up-regulated in PCa cell lines with NE features, neuroendocrine prostate cancer (NEPC) patient-derived xenograft models, and NEPC tissues.^53^

To evaluate whether SOX2 expression contributes to PCa progression, de Wet et al^24^ found that SOX2 expression did not independently promote an increase in tumor grade, nor did it significantly correlate with biochemical recurrence time, surgical margin status, pathological dissemination, local recurrence, or salvage therapy.^24^ However, SOX2-positive tumors exhibited significantly faster metastasis after biochemical recurrence than SOX2-negative tumors, and SOX2-positive patients had higher prostate cancer-specific mortality and overall mortality rates.^24^ Matsika et al^54^ also showed that among various prognostic parameters (such as lymphovascular invasion, extraprostatic extension, Gleason score, pathological lymph node staging, and pathological tumor staging), SOX2 was only significantly correlated with lymphovascular invasion.^54^ Recent studies further indicate that high SOX2 expression is negatively correlated with overall survival in PCa patients.^55^

In summary, SOX2 is primarily expressed in basal cells of normal and benign prostatic hyperplasia tissues, where it plays a role in maintaining the structural organization and self-renewal of prostate glands. However, SOX2 expression in primary PCa varies significantly. In mCRPC and NE tumors and at the invasive fronts of primary PCa, SOX2 expression is significantly elevated, which may contribute to the aggressiveness, migration, and therapy resistance of these tumors. This differential expression may be influenced by multiple factors, including patient characteristics, disease stage, treatment history, and the regulatory mechanisms governing tumor gene expression. For example, androgen signaling has been shown to suppress *SOX2* expression.^23^ Additionally, factors such as sample source, quantity, processing methods, antibody selection for SOX2 detection, and statistical thresholds used in data analysis may all impact the interpretation of SOX2 expression levels.

### SOX2 controls the fate of prostate cancer stem/progenitor cells

Cancer stem cells (CSCs), also referred to as tumor-initiating cells, possess the remarkable ability to reinitiate tumors and differentiate into various cell types when implanted into immunocompromised mice, thereby contributing to the significant cellular heterogeneity observed in primary tumors.^56^, ^57^, ^58^, ^59^ Similar to other solid tumors, PCa harbors CSCs, which exhibit a cell lineage hierarchy.^60^ Various molecular markers have been identified for isolating and characterizing prostate cancer stem cells (PCSCs), including cluster of differentiation 44 (CD44),^61^^,^^62^ CD133,^63^^,^^64^ α2β1 integrin,^65^^,^^66^ and aldehyde dehydrogenase (ALDH).^67^ As a critical stemness marker, SOX2 often works in conjunction with other factors such as ALDH, enhancer of zeste homolog 2 (EZH2), and octamer-binding transcription factor 4 (OCT4) to identify PCSCs and support their stem-like properties. Recently, Verma et al^68^ provided a comprehensive review of the identification, differentiation, and therapeutic responses of PCSCs.

Recent studies have demonstrated that non-tumorigenic cells can acquire stem-like properties, and cancer cells can reversibly and stochastically switch between tumorigenic and non-tumorigenic states.^69^^,^^70^ As one of the Yamanaka factors, including *c-MYC*, *SOX2*, *OCT4*, and Krüppel-like factor 4 (*KLF4*), SOX2 plays a pivotal role in generating induced pluripotent stem cells, in conjunction with other factors.^71^ For instance, Zhang et al^72^ successfully induced the formation of CSCs by transducing three factors (*OCT3/4*, *SOX2*, and *KLF4*) into PCa cells. This innovative approach not only facilitated the large-scale acquisition of prostate tumor-initiating cells but also provided deeper insights into their biological characteristics, paving the way for developing novel therapies targeting prostate tumor-initiating cells. Similarly, Vêncio et al^73^ used lentiviral vectors carrying POU class 5 homeobox 1 (*POU5F1*), *LIN28*, *NANOG*, and *SOX2* to transfect CD90^+^ PCa-associated stromal cells—a distinct pathological cell type exclusive to tumor tissues—successfully generating alkaline phosphatase-positive induced pluripotent stem cells. These stem cells exhibited gene expression profiles and clonal morphologies closely resembling those of human embryonic stem cells, highlighting their potential as a model system for studying PCSC biology and therapies.

In embryonic stem cells, SOX2, NANOG, and OCT4 form a core regulatory network that maintains the expression of pluripotency-associated genes.^74^^,^^75^ Similarly, SOX2 can cooperate with OCT4 to regulate the pluripotency of PCa cells.^76^ However, due to tumor cell heterogeneity, SOX2 exerts stemness-regulating effects in certain PCa cell lines. Sotomayor et al^77^ found that SOX2 expression was detected in the OCT4A-expressing cell subpopulation within PCa tissues; however, these cells lacked proliferative capacity and did not express other stemness markers such as ATP binding cassette subfamily G member 2 (ABCG2), NANOG, or CD133.^77^ Srinivasan et al^78^ have pointed out that while SOX2 serves as a presumed stemness marker in PC3 cells, it does not exhibit similar functions in DU145, PCa2b, or LNCaP cells. Additionally, SOX2 knockdown did not significantly affect the expression levels of OCT4 and NANOG, but markedly reduced the number of tumor spheres formed.^78^ On the other hand, SOX2 may interact with tumor-specific non-embryonic chaperone proteins in cancer cells to regulate a novel set of genes, executing unique non-stemness biological functions. In PrEC and CWR cell lines, neither NANOG nor OCT4 expression was detected, and SOX2 expression was not associated with the up-regulation of stemness markers, enhancement of tumor sphere-forming ability, or induction of embryonic stem cell-related SOX2 target gene expression^23^ Further, chromatin immunoprecipitation-sequencing analysis for SOX2 in embryonic stem cells and CWR cells revealed that the primary gene targets regulated by SOX2 in PCa cells were almost entirely distinct from its targets in embryonic stem cells. Even for the same gene, SOX2 binding sites differ significantly. These findings highlight the functional complexity of SOX2 in PCa cells, reflecting the high degree of heterogeneity in PCa.^24^

## SOX2 regulates malignant biological behaviors

### PCa cell proliferation and apoptosis resistance

Jia et al^49^ demonstrated that *SOX2* overexpression significantly promoted PCa cell proliferation *in vitro*, increased the proportion of cells in the S-phase, and enhanced resistance to chemotherapy-induced apoptosis. *In vivo* experiments further supported these findings, showing that SOX2 down-regulation significantly inhibited tumor growth and enhanced the sensitivity of tumors to chemotherapy. Mechanistically, SOX2 modulates calcium signaling by down-regulating store-operated Ca^2+^ entry and reducing calcium release-activated calcium modulator 1 (Orai1) protein expression, which in turn impacts apoptosis regulation. Chen et al^79^ extended this understanding by showing that SOX2 could regulate the cell cycle and promote PCa tumorigenesis through its interaction with chloride channel voltage-gated 3 (CLC-3). Inhibition of either CLC-3 or SOX2 led to cell cycle arrest at the G0/G1 phase, accompanied by a significant decrease in cyclin D1 protein levels and a corresponding increase in P27 protein levels.^79^ Interestingly, Metz et al^80^ found that elevated SOX2 induced a reversible growth arrest state, both *in vivo* and *in vitro*, conferring resistance to conventional chemotherapy in tumor cells. This effect is associated with SOX2 lowering cyclin and cyclin-dependent kinase levels, while simultaneously up-regulating the expression of p27^Kip180^. These findings further underscore the critical role of SOX2 in regulating PCa cell proliferation and apoptosis resistance.

### Tumor metastasis in PCa

Tumor metastasis remains a major challenge in PCa treatment, accelerating disease progression and reducing the effectiveness of therapies. Metastasis is a complex, multi-step process, with invasion being a key early step often triggered by epithelial–mesenchymal transition (EMT).^81^^,^^82^ Studies have shown that *SOX2* expression is up-regulated in highly invasive cells, with its presence being crucial for their invasiveness. Targeted knockout of *SOX2* significantly inhibited the invasiveness of PCa cells.^83^ During EMT, epithelial cells progressively lose their epithelial characteristics and markers (*e.g.*, E-cadherin), while up-regulating the expression of EMT regulators (*e.g.*, Snail, Twist, Zeb-1/2) and mesenchymal markers. These changes endow the cells with enhanced migration and invasive capabilities.^84^ SOX2 has been shown to regulate these EMT-related factors. Specifically, the downstream genes *SNAI1/Snail*, *TWIST2*, and zinc finger E-box binding homeobox 2 (*ZEB2*) are significantly up-regulated in *SOX2*-overexpressing cells, further promoting the invasive phenotype of PCa cells.^22^ Additionally, SOX2 modulates the expression of various growth factors, angiogenesis factors, and lymphangiogenesis factors, such as neuropilin-2 (NRP2), fibroblast growth factor 2 (FGF2), and vascular endothelial growth factor C (VEGFC), thereby promoting the ability of PCa cells to enter the circulatory system, which is a key step in metastasis.^22^ However, once cancer cells enter the bloodstream, only a small proportion, often CSCs, can survive and colonize secondary sites.^85^, ^86^, ^87^ The role of SOX2 in maintaining CSC survival and multipotency highlights its crucial function in the later stages of metastasis, particularly in supporting the adaptability of metastatic tumor cells to new microenvironments.^24^

In conclusion, SOX2 plays a critical role in both the progression and metastasis by regulating cell proliferation, apoptosis resistance, and invasive capacity. Its involvement in the EMT process and regulation of key downstream genes significantly contribute to the invasive and metastatic potential of PCa cells. Furthermore, SOX2's role in maintaining CSCs suggests that it is a key factor in the late stages of metastasis, ensuring the survival and growth of metastatic tumor cells. These findings emphasize the potential of targeting SOX2 in therapeutic strategies aimed at controlling PCa progression and metastasis.

### SOX2 promotes lineage plasticity and induces NE phenotype in PCa

Next-generation androgen receptor pathway inhibitors, such as abiraterone and enzalutamide, have significantly extended survival in patients with mCRPC.^88^^,^^89^ However, long-term use of these drugs inevitably leads to resistance development. Unlike traditional androgen deprivation therapy resistance mechanisms, resistance to androgen receptor pathway inhibitors primarily arises through lineage plasticity mechanisms.^90^, ^91^, ^92^ Lineage plasticity enables PCa cells to undergo lineage reprogramming under therapeutic pressure, thereby generating alternative cell populations with resistance traits. The most common of these is NEPC.^93^

**Figure 1:**
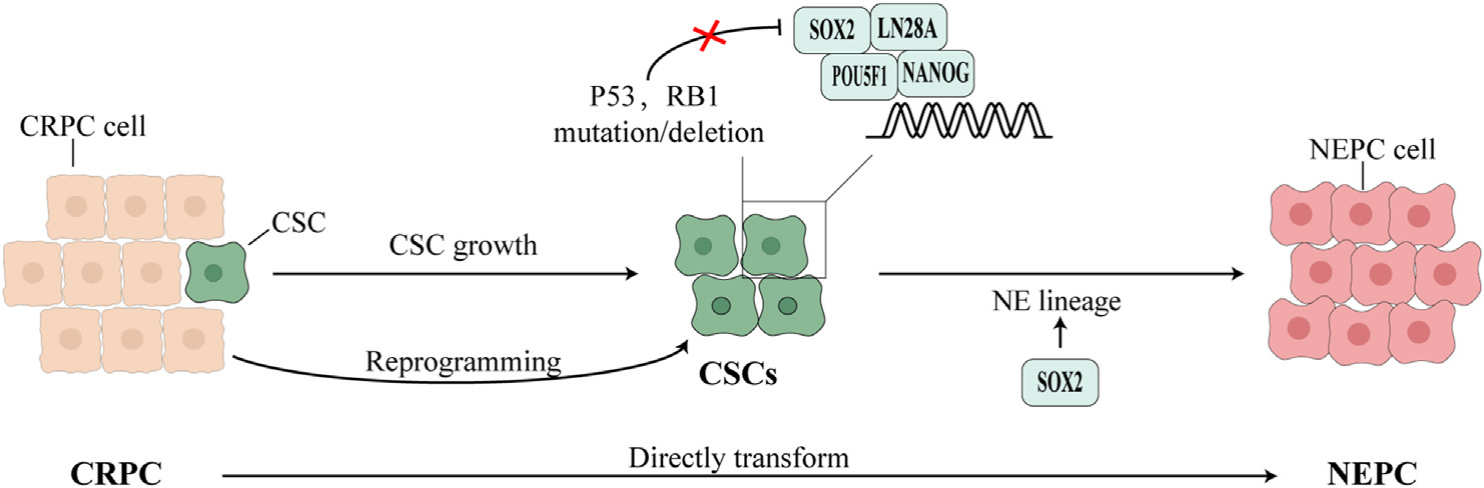
The role of SOX2 in lineage conversion from CRPC to NEPC. The growth of CSCs and the reprogramming of non-CSCs contribute to an intermediate stem cell-like state, which is maintained and regulated by SOX2 and other pluripotency factors. Subsequently, SOX2 drives the conversion of CRPC into NEPC by inducing the transition to the NE lineage. Furthermore, CRPC can directly transform into NEPC without necessarily passing through the intermediate stem cell-like state. CRPC, castration-resistant prostate cancer; NEPC, neuroendocrine prostate cancer; CSC, cancer stem cell; NE, neuroendocrine.

NEPC formation involves two major mechanisms: first, the malignant transformation of normal NE cells surrounding CRPC^94^; and second, the lineage conversion of adenocarcinoma cells into NEPC through genetic and epigenetic changes.^95^ Tumor cells undergoing treatment-induced lineage conversion typically revert to a stem-cell-like intermediate state, which subsequently differentiates into NEPC. These intermediate-state CSCs may originate from pre-existing CSCs in CRPC or from AR-dependent luminal epithelial cells that undergo reprogramming (Fig. 1). Notably, NEPC and AR-positive adenocarcinoma cells exhibit highly similar genomic features, suggesting that androgen receptor pathway inhibitor-induced NEPC is mainly driven by lineage plasticity.^93^^,^^96^ This section explores the role of SOX2 in the transition from CRPC to NEPC and its underlying mechanisms.

### SOX2 mediates the induction and regulation of stem-cell-like intermediate states

Stem-cell-like intermediate states provide PCa cells with multi-lineage differentiation potential, enabling them to rapidly adapt under therapeutic stress and transform into more survival-advantageous phenotypes.^92^ Cells in these intermediate states typically exhibit down-regulated expression of Rb1 and p53 proteins, along with aberrant overexpression of SOX2 and SOX9.^97^ Gene-engineered mouse models suggest that this stem-cell-like epigenetic environment promoting lineage plasticity is mainly driven by epigenetic reprogramming factors such as Sox2 and EZH2.^91^ These factors drive AR-dependent luminal epithelial cells to transition into non-AR-dependent basal-like cells. Importantly, the reversibility of tumor cell plasticity means that this process can be reversed by restoring TP53 and RB1 function or inhibiting SOX2 expression^91^ (Fig. 1). Moreover, this intermediate cell state is maintained by a gene network composed of various stemness factors, with SOX2 playing a central role. For example, Lovnicki et al^98^ analyzed tumor samples from NEPC patients, patient-derived xenograft models, transgenic mice, and cell models, finding that lin-28 homolog B (LIN28B) up-regulation could activate the stem-cell-like gene network via the Lin28B/let-7/SOX2 axis, driving the expression of NE markers and promoting NEPC progression.^98^ Furthermore, the RNA splicing factor SRRM4 has been shown to induce the SOX2-mediated pluripotency gene network, playing a critical role in NEPC development.^99^

Further studies emphasize the importance of the sequential activation of stem-cell transcription factors in PCa progression. Liu et al^100^ demonstrated that during the transition from adenocarcinoma to undifferentiated small-cell carcinoma, POU5F1, lin-28 homolog A (LIN28A), SOX2, and NANOG are sequentially activated, driving the transformation of differentiated adenocarcinoma to undifferentiated small-cell carcinoma. Prostate stromal proenkephalin has been shown to suppress the expression of these factors, promoting the transition of cancer cells into a more differentiated state.^100^ These findings underscore the central role of the stem-cell-like state in treatment-induced lineage plasticity. Thus, high expression of POU5F1, ALDH1, and SOX2 in enzalutamide-resistant cells and samples from androgen deprivation therapy-treated patients may signify a stem-cell-like state, granting tumor cells significant lineage plasticity and enabling them to evade immune and targeted therapies.^101^

### SOX2 induces NE phenotypes

When treatment-resistant, intermediate stem cell-like cancer cells receive NE lineage-related signals and activate NE gene expression programs, adenocarcinoma can convert into NEPC. This transformation is regulated by a multi-layered network, including transcription factors, epigenetic modifications, key signaling pathways, and the tumor microenvironment. Single-cell RNA sequencing studies have identified several transcription factor regulatory networks driving the conversion from adenocarcinoma to NEPC. These networks involve the constitutive regulation of achaete-scute complex-like 1 (*ASCL1*) and forkhead box A2 (*FOXA2*), along with the selective regulation of NK2 homeobox 2 (*NKX2-2*), POU class 3 homeobox 2 (*POU3F2*), and *SOX2*.^102^ Together, these factors drive the formation of NEPC. In this section, we focus on SOX2, examining its upstream regulatory network and downstream effects.

Several molecules regulate *SOX2* expression, which is essential for the development of NEPC. One key regulator is the neural transcription factor BRN2 (encoded by POU3F2), which is significantly up-regulated in NEPC tissues. Although its expression is suppressed by AR signaling, BRN2 can directly bind to the enhancer region of *SOX2*, thereby promoting its expression. The genes co-regulated by BRN2 and SOX2 in neural progenitor cells are also enriched in NEPC tissue samples, suggesting that BRN2 and SOX2 work synergistically to promote NEPC formation. Notably, in prostate cancer cells where *BRN2* is silenced and *SOX2* is overexpressed, NE marker expression does not increase. This suggests that SOX2-dependent neuroendocrine differentiation requires *BRN2* participation.^53^ *BRN2* is itself regulated by various upstream signals. For instance, in MUC1-C-mediated prostate cancer cell lineage plasticity models, MUC1-C forms a complex with MYC and binds to the *BRN2* promoter region. This up-regulates both *BRN2* and *SOX2* expression, thereby promoting the conversion of CRPC to NEPC.^103^ Additionally, the pseudokinase tribbles 2 (TRIB2) is significantly up-regulated in enzalutamide-resistant prostate cancer cells and tumors. TRIB2 activates *BRN2* and *SOX2* while suppressing AR and cytokeratin 8 (CK8), thus inducing the NE phenotype. Importantly, inhibiting *TRIB2* or its downstream targets, *BRN2* and *SOX2*, restores the sensitivity of resistant cells to enzalutamide.^104^

In tumor organoid models, it was found that TACSTD2 and SOX2 formed a positive feedback loop in TACSTD2^high^ luminal cells, together driving neuroendocrine differentiation and castration resistance.^105^ Additionally, NRP2 interacts with VEGFR2 through its sea-urchin sperm protein, enterokinase, and agrin (SEA) domain, thereby activating signal transducer and activator of transcription 3 (STAT3) phosphorylation. This activation promotes SOX2 expression, enhances NEPC invasiveness, and induces the expression of NE markers. Inhibiting the VEGFR2/STAT3/SOX2 axis significantly reduces NEPC occurrence and progression.^106^ Moreover, AR can directly bind to the promoter of tripartite motif containing 59 **(***TRIM59*), thereby suppressing its expression. Androgen receptor pathway inhibitor treatment can relieve the transcriptional repression of *TRIM59*, leading to its up-regulation. This up-regulation, in turn, promotes the degradation of RB1 and P53, enhancing SOX2 expression and driving NEPC development.^107^

SOX2 plays a key role in regulating NEPC-associated gene expression through multiple mechanisms, maintaining the NE phenotype. *In vitro* cell model studies have shown that SOX2 activates lysine-specific demethylase 1 (LSD1), significantly inhibiting the expression of adenocarcinoma-specific genes through epigenetic regulation, while mildly inducing the expression of certain NE markers. This dual role enhances the NE phenotype and drives NEPC progression.^108^ Gene-engineered mouse models further confirm that Sox2 is essential for neuroendocrine differentiation in phosphatase and tensin homolog (Pten)-deficient prostate adenocarcinomas induced by treatment. Interestingly, Sox2 does not affect castration-resistant characteristics,^109^ aligning with previous studies.^110^

In addition to these effects, high expression of SOX2 is closely associated with the transcriptional activation of lncRNA H19. H19 regulates the expression of AR target genes and NEPC-related genes by promoting methylation modifications at H3K27me3/H3K4me3 histone sites, regulated by polycomb repressive complex 2 (PRC2). H19 also induces DNA methylation changes at CpG sites across the genome, further promoting the transcription of NEPC-related genes. SOX2 directly binds to the H19 promoter, enhancing its transcription and forming a positive feedback loop with H19, jointly driving NEPC progression.^111^

SOX2 also plays a key role in the activation of serine peptidase inhibitor Kazal type 1 (*SPINK1*) transcription, which supports the NE phenotype. Pharmacological inhibition of casein kinase-1 (CK1) stabilizes repressor element-1 silencing transcription factor (REST), which, in synergy with AR signaling, suppresses SPINK1 expression and blocks SOX2-mediated NEPC progression.^112^ Additionally, SOX2 enhances the transcriptional activity of *ASCL1*, a key player in neuronal differentiation and positioning. ASCL1 is highly expressed in NEPC cells and is associated with poor prognosis. Mechanistically, ASCL1 promotes the phosphorylation of cAMP response element-binding protein 1 (CREB1), inhibiting ferroptosis and promoting NEPC cell survival, thus driving NEPC formation.^113^

In summary, SOX2 is central to the initiation and progression of NEPC, functioning through a multi-layered regulatory network. Its upstream regulation involves factors such as BRN2, TRIB2, NRP2, and TRIM59, while its downstream effects are mediated through key pathways like LSD1, H19, SPINK1, and ASCL1, all of which maintain the NEPC phenotype. Further exploration of the molecular mechanisms governing SOX2 and its associated regulatory networks could reveal new diagnostic and therapeutic strategies for NEPC. Developing inhibitors targeting SOX2 and its signaling pathways may offer promising therapeutic avenues to improve NEPC prognosis.

### Regulation of SOX2 expression in prostate cancer

Multiple studies have revealed the molecular regulatory mechanisms of *SOX2* in various cancers, but our understanding of its specific role in PCa progression remains limited. This part of the review focuses on the regulation of *SOX2* expression in the following aspects: i) other transcription factors; ii) non-coding RNAs (mainly miRNAs and lncRNAs); and iii) epigenetic mechanisms that affect its expression.

#### Regulation by transcription factors

Although SOX2 is a transcription factor, its expression is regulated by several other transcription factors. In addition to the factors mentioned in the previous section, which induce the NE phenotype by regulating *SOX2*, there are other transcription factors that modulate its transcriptional activity by interacting with specific binding sites in the *SOX2* promoter region. For example, AR has been well established as a transcriptional activator in the progression of PCa. In addition to its traditional tumor-promoting effects, studies have also identified that AR possesses tumor suppressor functions.^114^^,^^115^ In PCa cell lines, AR directly inhibits *SOX2* expression by binding to enhancer elements in the *SOX2* promoter, thus exerting a transcriptional repressive effect.^23^ Similarly, in normal prostate epithelial cells and human embryonic stem cells, enhanced AR signaling reduces *SOX2* expression.^23^ In CRPC cells, *SOX2* expression can be induced by androgen deprivation (*e.g.*, enzalutamide), whereas the synthetic androgen R1881 suppresses *SOX2* expression.^23^^,^^53^

Esposito et al^116^ reported that *SNAI2* expression was down-regulated in most PCa cells but up-regulated in cell clusters at the expansion/invasion front, the neuroendocrine differentiation region, and lymph node metastases. SNAI2 regulates pluripotency genes, including *SOX2*. Lee et al^117^ found that SLUG/SNAI2 stabilized *SOX2* by interacting with it and preventing its proteasomal degradation, while TWIST1 up-regulated *SOX2* transcription by binding to the proximal E-box element in the *SOX2* promoter. Together, these factors work synergistically to maintain PCSC characteristics. Interestingly, there is a cross-regulatory mechanism between *SNAI2/SLUG* and *SOX2*. Down-regulation of *SOX2* not only decreases the expression of *SNAI2/SLUG* but also inhibits the migration and tumor sphere formation of PC3 cells.^78^ Additionally, hypoxia-inducible factors (HIF-1/2α) play a role in regulating *SOX2* expression. Acute and chronic hypoxia increase the levels of HIF-1α and HIF-2α, respectively, which up-regulate *SOX2* expression. Loss of SOX2 impairs hypoxia-induced cellular functions, such as migration and invasion.^118^

#### Regulation by miRNA and lncRNA

miRNA is a class of small non-coding RNAs, approximately 19–24 nucleotides long, that play an essential role in post-transcriptional regulation of mRNA expression.^119^^,^^120^ In PCa, the expression of several miRNAs is dysregulated compared with normal epithelial tissues, with some of these miRNAs exhibiting carcinogenic or tumor suppressor effects during the occurrence and progression of the disease.^121^^,^^122^ Forno et al^123^ reported that the loss of miRNA-34b expression occurred selectively in hormone-dependent PCa. This loss is associated with epigenetic silencing of the MIR34 B/C site and increased DNA copy number loss. Down-regulation of miR-34b relieves its inhibition of downstream target genes, leading to the overexpression of *SOX2*. These findings suggest that miR-34b could serve as a potent biomarker for predicting the progression of hormone-sensitive PCa.

Ozen et al^124^ demonstrated the tumor-suppressive function of miR-145-5p. In human PCa cell lines. *miR-145-5p* expression was significantly down-regulated, and ectopic expression of miR-145-5p inhibited cell proliferation and migration, induced apoptosis, and up-regulated *SOX2* expression. However, this study did not definitively confirm *SOX2* as a direct target gene of miR-145-5p. Additionally, Tohidast et al^125^ further corroborated the inhibitory effects of miR-145 on PCa cell migration and its role in increasing paclitaxel chemosensitivity. They found that both miR-145 and paclitaxel alone significantly reduced the expression of *CD44* and *SOX2*, with the combination treatment proving more effective than either treatment alone in reducing *CD44* and *SOX2* expression. These results highlight the potential therapeutic value of miR-145 in PCa treatment.^125^

In addition to miR-145, miR-126 and miR-149 also play crucial roles in PCa progression. Studies have shown that these two miRNAs act as oncogenic miRNAs by targeting *SOX2*, and their expression levels are significantly down-regulated in PCa tissues.^126^ The miR-200 family is instrumental in regulating PCSC characteristics and EMT phenotypes. Specifically, miR-200b and miR-200c negatively regulate the expression of *SOX2* and other EMT-related genes, linking CSC characteristics with EMT phenotypes. Studies have shown that re-expression of miR-200b and miR-200c can reverse EMT to mesenchymal–epithelial transition phenotypes, significantly inhibiting the sphere-forming ability of PCa cells with EMT characteristics.^127^ Furthermore, the loss of miR-200c-3p expression is closely associated with the regulation of T-box transcription factor 2 (TBX2) in CRPC. TBX2 binds to the *miR-200c-3p* promoter, inhibiting its expression, and blocking TBX2 can restore miR-200c-3p expression. This study highlights miR-200c-3p as a key intermediate effector of TBX2 in regulating *SOX2* and *N-MYC* expression.^128^

lncRNAs also play a critical role in regulating SOX2 expression. For instance, PIM1, a member of the PIM protein kinase family, induces the expression of lncRNA H19, which in turn activates the transcription of *SOX2*, *NANOG*, and *OCT4*. Studies have shown that small-molecule pan-PIM inhibitors can significantly down-regulate H19 expression, leading to a reduction in SOX2 levels and enhancing the sensitivity of LNCaP cells to enzalutamide. Notably, this sensitivity can be reversed by overexpressing H19, which is associated with up-regulation of SOX2 expression, thereby enhancing cellular stemness and promoting resistance to androgen deprivation therapy.^129^

The role of non-coding RNAs in various cancers, including PCa, has been extensively studied. However, most studies focused on the regulatory relationship between non-coding RNAs and *SOX2*, while research on their underlying molecular regulatory mechanisms remains relatively limited. Therefore, further investigation into the molecular mechanisms governing non-coding RNA regulation will provide a crucial foundation for developing therapeutic strategies targeting non-coding RNAs.

#### Epigenetic regulation of SOX2 expression

Epigenetic modifications of DNA and histones are crucial for regulating proper gene expression patterns. Research indicates that abnormal epigenetic alterations are common in various cancers, including PCa, and may lead to dysregulated SOX2 expression, subsequently activating oncogenes or inhibiting tumor suppressor genes.^130^^,^^131^

Methylation of the SOX2 promoter can result in its down-regulation in PCa samples, effectively achieving epigenetic gene silencing. However, Kar et al^132^ point out that DNA promoter methylation is not a key regulator of SOX2 expression in PCa cell lines. Instead, they emphasize that histone modifications play a more significant role in regulating SOX2 expression. Specifically, the accumulation of H3K4me3 and H3K9acS10p, combined with a sharp decrease of H3K9me3 and H3K27me3, contributes to the activation of the SOX2 gene promoter.

### SOX2-mediated signaling pathways in prostate cancer

The complex signaling network involves numerous molecules that interact with SOX2 to regulate various biological processes in tumor cells, promoting malignant tumor progression.

#### Hedgehog signaling pathway

SOX2 and the Hedgehog signaling pathway act synergistically to promote PCa progression. Simultaneous targeting of both SOX2 and the Hedgehog pathway inhibits cell proliferation, increases apoptosis, and reduces cell migration.^133^ The Hedgehog pathway also regulates *SOX2* expression. In PCa cells treated with cyclopamine, an inhibitor of the Hedgehog pathway receptor SMO, *SOX2* expression was greatly down-regulated.^133^ Further studies have shown that palmitate and cholesterol promote *SOX2* expression in PCa cells by activating the Hedgehog pathway. Zhang et al^134^ extended these results, indicating that palmitate and cholesterol could promote SOX2 expression in PCa cells by activating the Hedgehog signaling pathway. When co-cultured with cancer-associated fibroblasts, palmitate's effect on SOX2 expression was further enhanced. Mechanistically, Wnt5a derived from cancer-associated fibroblasts promotes Hedgehog-mediated SOX2 expression in PCa cells, showing that palmitate and cholesterol induce SOX2 expression through cancer–stromal interactions involving Hedgehog signaling and non-canonical Wnt signaling.

#### PI3K/AKT signaling pathway

Activation of the phosphatidylinositol 3-kinase (PI3K)/protein kinase B (AKT) signaling pathway plays a critical role in maintaining *SOX2* expression. Vaddi et al^76^ demonstrated that in human PCa cell lines with SOX2/OCT4 overexpression (SORE6^+^), the phosphorylation level of AKT was significantly elevated compared with SORE6-cells, although the total AKT level and the catalytic subunits of PI3K, p110α and p110β, remained unchanged. Treatment with inhibitors targeting specific PI3K/AKT signaling components significantly reduced the proportion of SORE6^+^ cells. Gene set enrichment analysis further revealed that the mammalian target of rapamycin (mTOR), Kirsten rat sarcoma viral oncogene homologue (KRAS), Erb-B2 receptor tyrosine kinase 2 (ERBB2), and Wnt signaling pathways were significantly up-regulated, while the PTEN pathway was notably down-regulated in SORE6^+^ cells. Lin et al^135^ found that inhibitors of the PI3K/AKT pathway effectively reversed the up-regulation of SOX2 and survivin protein expression induced by transforming growth factor alpha (TGF-α). Additionally, SOX2 not only promotes cell cycle progression by regulating cell cycle-related proteins but may also influence apoptosis in PCa cells through the regulation of survivin.

#### TGF-β and Wnt signaling pathway

The poor prognosis of PCa is often associated with the high invasiveness and metastatic potential of tumor cells. Li et al^136^ demonstrated that transforming growth factor beta (TGF-β) signaling enhances the invasiveness of SOX2-driven tumor cells, a process potentially mediated through the induction of EMT. However, compared with TGF-β signaling, the Wnt/β-catenin signaling pathway plays a more prominent role in SOX2-mediated PCa metastasis. Further studies have shown that in breast cancer MDA231 cells, SOX2 directly interacts with the promoter region of β-catenin to regulate its expression. In both breast and PCa cell lines, SOX2 further modulates the activity of the WNT signaling pathway by regulating key regulators or downstream effector molecules of the pathway, such as dickkopf WNT signaling pathway inhibitor 3 (DKK3), dishevelled-1 (DVL1), and DVL3.

#### Other signaling pathways

**Figure 2:**
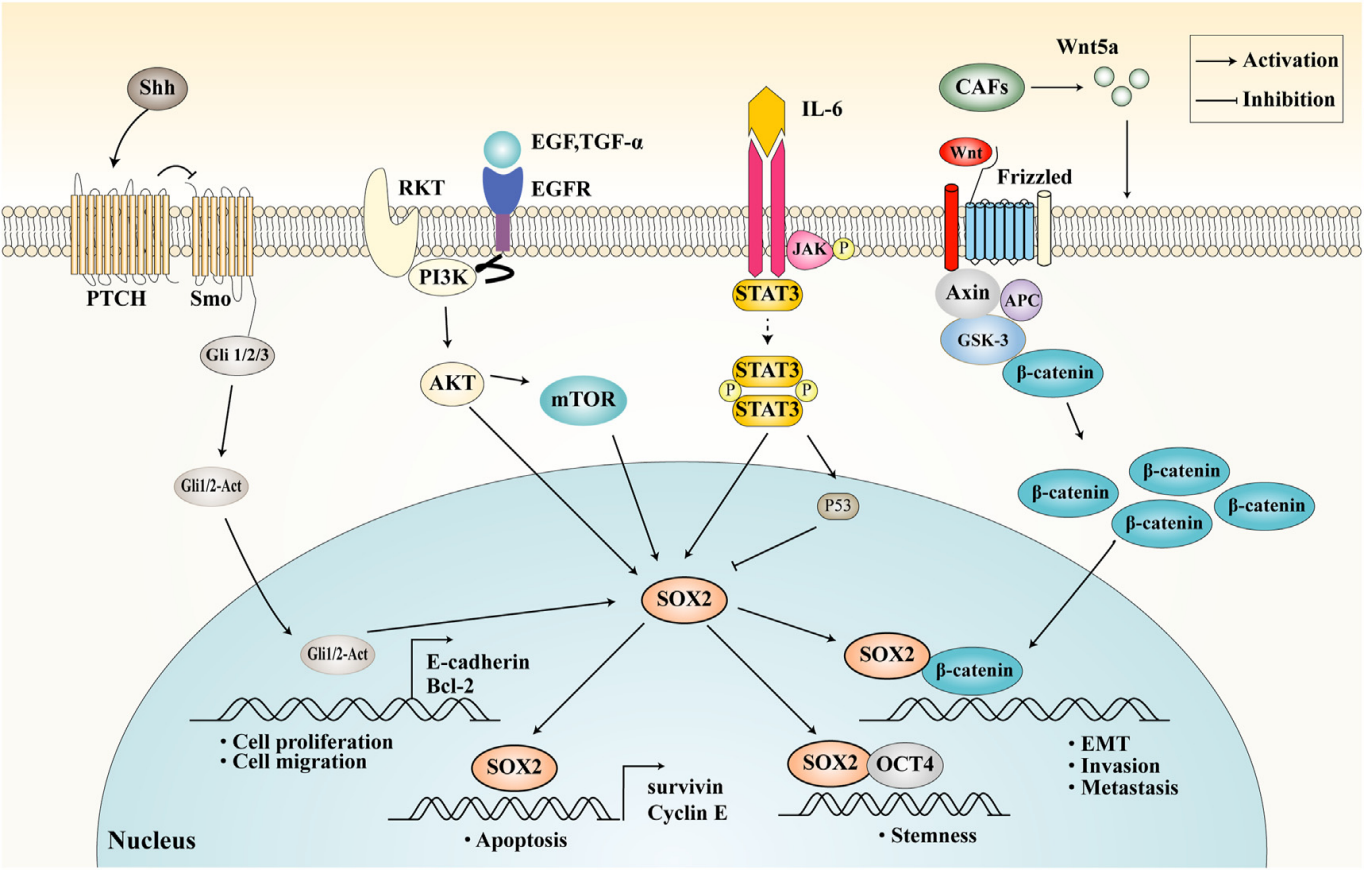
The SOX2-regulated signaling network drives prostate cancer progression. Data in this figure were obtained using androgen receptor (AR)-negative models, which more accurately represent signaling in advanced AR-therapy resistant prostate cancer cells. The synergy of SOX2 with the Hedgehog (HH) pathway promotes prostate cancer progression. Simultaneous inhibition of the SOX2 and HH pathways can significantly reduce cell proliferation and migration, decrease the expression of the epithelial–mesenchymal transition (EMT) marker E-cadherin and the anti-apoptotic protein Bcl-2, and increase the expression of the apoptotic protein Bax. The HH signaling pathway also regulates *SOX2* expression. Palmitic acid and cholesterol up-regulate *SOX2* through the HH signaling pathway, a process enhanced by Wnt5a derived from cancer-associated fibroblasts. The PI3K/AKT pathway is critical for maintaining *SOX2* expression. TGF-α can activate the PI3K/AKT pathway, thereby inducing the expression of SOX2 and survivin. SOX2 can bind to the β-catenin promoter, promoting tumor EMT, invasion, and metastasis. Additionally, EGFR signaling up-regulates *SOX2* in prostate cancer stem cells, and EGFR inhibitors can block this effect. Alanolactone inhibits IL-6-induced STAT3 signaling, down-regulates SOX2, and up-regulates P53.

Multiple signaling pathways play a critical role in maintaining and regulating PCa stemness by modulating SOX2 expression and function. In PC3 cells, knockdown of *WNT10B* reduced *SOX2* and *NANOG* expression, as well as the proportion of stem cell-like side populations.^137^ Furthermore, the epidermal growth factor receptor (EGFR) signaling pathway positively regulates *SOX2* expression in PCSCs. Studies have shown that EGFR inhibitors block the up-regulation of *SOX2* expression and the enhancement of self-renewal capacity induced by EGFR activation.^138^ Additionally, the compound alanolactone inhibits interleukin-6 (IL-6)-induced STAT3 phosphorylation, down-regulates the expression of SOX2, OCT4, NANOG, CD133, and CD44, and up-regulates P53 expression, modulating the stemness of PCa cells.^139^ A diagram of multiple interactive signaling pathways involving SOX2 in PCa is shown in Figure 2.

### The role of SOX2 in the treatment resistance of prostate cancer

**Figure 3:**
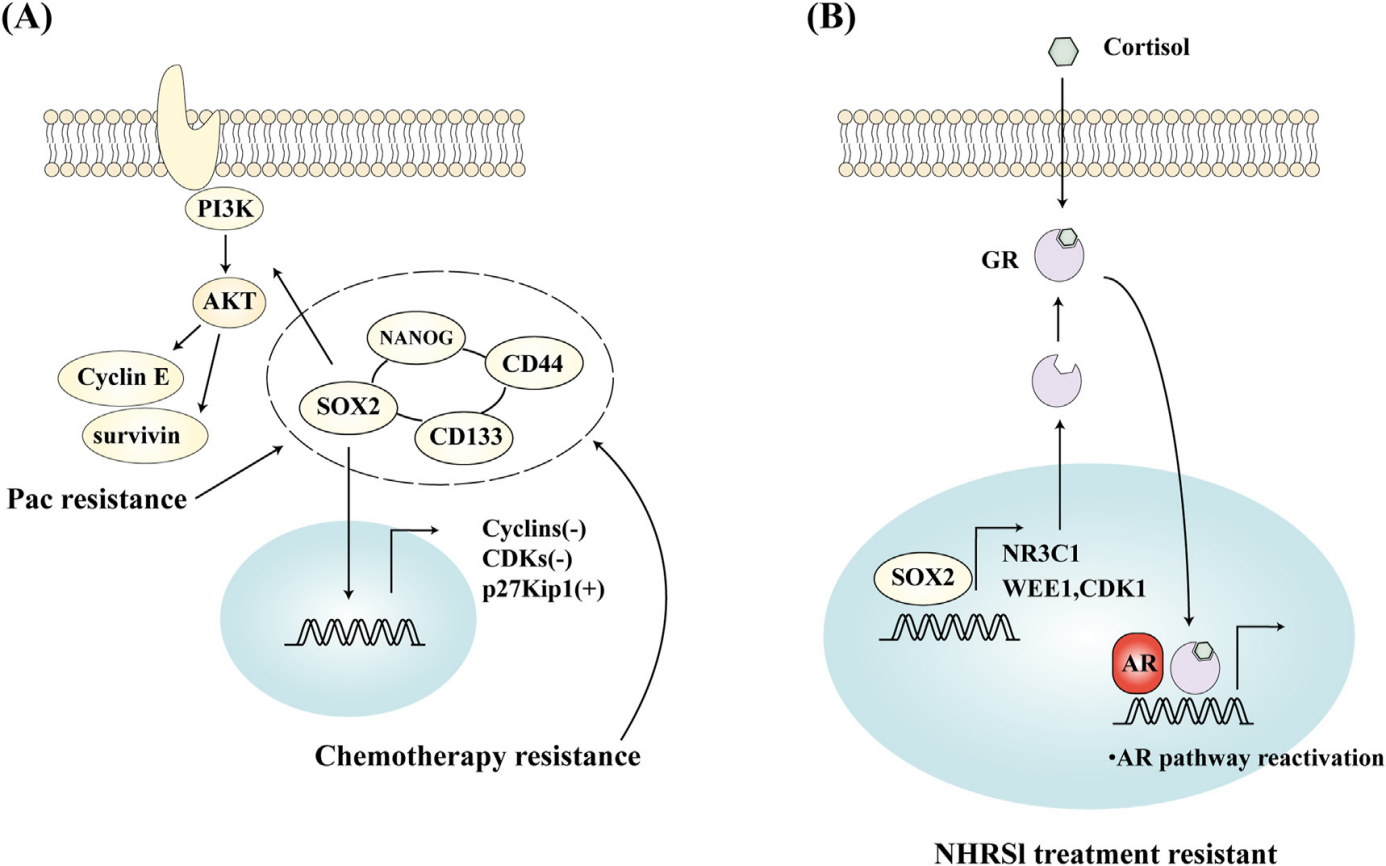
SOX2 mediates chemotherapy and nuclear hormone receptor signaling inhibitor (NHRSI) resistance. **(A)** Chemotherapy resistance: SOX2 induces a “quiescent state” in cells and activates the PI3K/AKT pathway to resist chemotherapy. Conversely, drug resistance also leads to the enrichment of stemness markers such as SOX2. **(B)** NHRSI resistance: SOX2 transcriptionally activates *NR3C1* to up-regulate glucocorticoid receptor (GR), and GR activation restores androgen receptor (AR) downstream signaling.

PCSCs are closely linked to resistance to radiotherapy and chemotherapy, aggressive cancer behavior, and increased recurrence rates.^140^^,^^141^ In contrast, radiotherapy, chemotherapy, and endocrine therapy can induce a drug-resistant phenotype in tumor cells and enhance the functional enrichment of PCSCs.^142^ For instance, in non-tumorigenic PCa cells, treatment with docetaxel reprograms these cells to adopt a PCSC phenotype, enhancing their ability to form prostate spheres^143^ (Fig. 3A). Furthermore, overexpression of *SOX2* in the PC-3 cell line significantly increases resistance to paclitaxel, mediated by the sustained activation of the PI3K/Akt signaling pathway driven by SOX2. SOX2 targets cyclin E and survivin, promoting cell proliferation and inhibiting apoptosis^144^ (Fig. 3A). In addition, SOX2 regulates a distinct cellular metabolic phenotype in PCa, with its expression positively correlating with enhanced glycolytic activity, basal respiration, maximal respiration, and spare respiratory capacity. Cells expressing SOX2 exhibit higher mitochondrial respiration and glycolytic capacity, which collectively bolster the therapeutic resistance and plasticity of SOX2-positive PCa cells.^24^

### SOX2 is implicated in mediating resistance to nuclear hormone receptor signaling inhibitors (NHRSIs)

**Figure 4:**
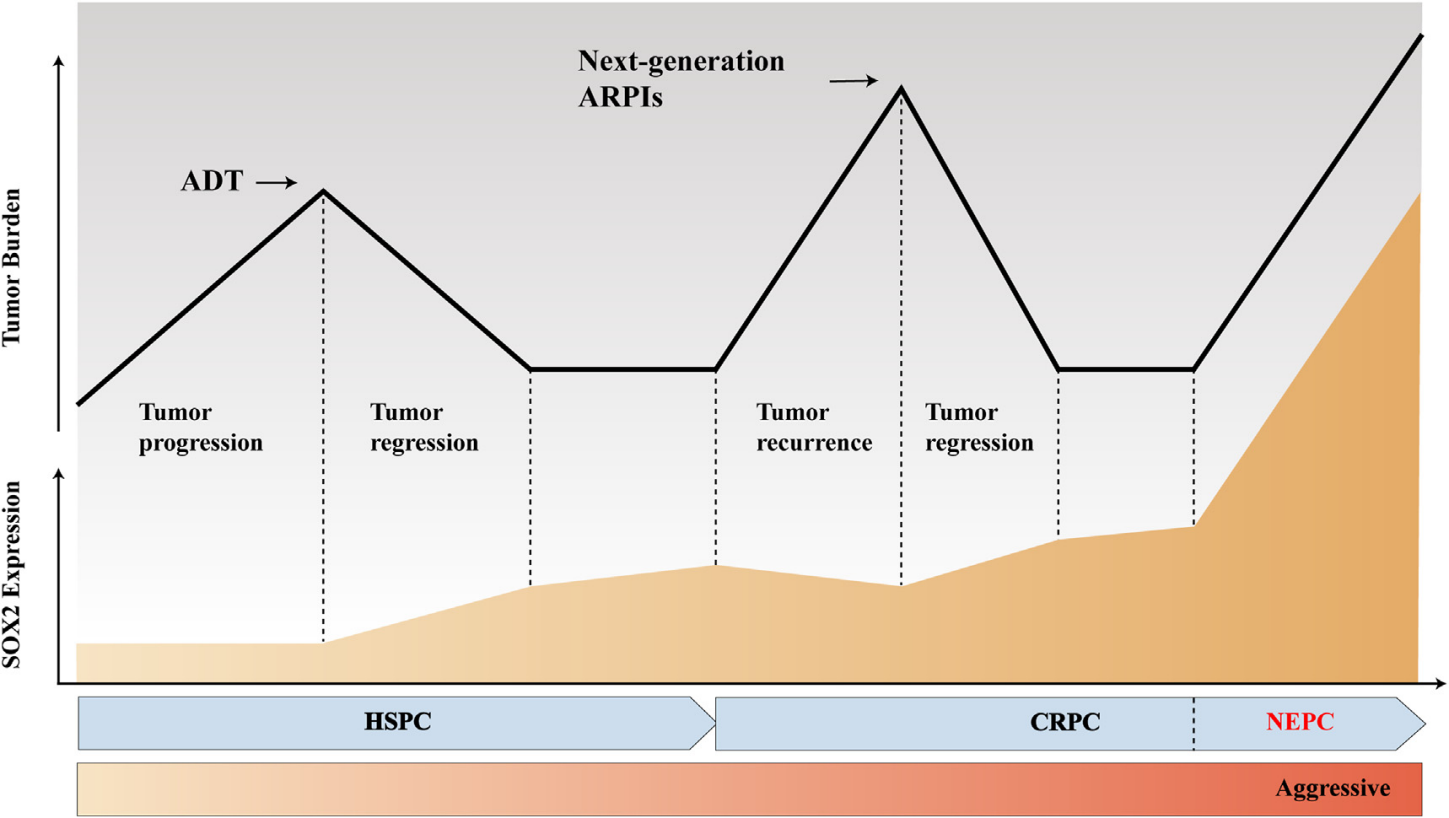
The process of prostate cancer resistance to endocrine therapy and its relation to *SOX2* expression (orange area). During the castration-sensitive stage, *SOX2* is a target gene of the androgen receptor (AR). Continuous inhibition of the AR leads to increased *SOX2* mRNA expression levels and decreased prostate-specific antigen (PSA) expression. However, with *AR* mutation and bypass activation, prostate cancer enters the castration-resistant stage. At this stage, PSA expression increases, and *SOX2* expression is no longer completely dependent on AR regulation. *SOX2* expression continues to be up-regulated, thereby enhancing the lineage plasticity of prostate cancer, causing it to lose its dependence on AR completely.

PCa is a hormone-dependent tumor that relies on AR signaling to sustain tumor growth in its early stages. Androgen deprivation therapy effectively reduces androgen levels and/or directly blocks AR, but metastatic PCa generally develops resistance. This resistance is usually due to various mechanisms that reactivate AR-mediated signaling, including genomic mutations, amplifications, and AR splice variants.^145^^,^^146^ Activation of bypass pathways also plays a significant role. After androgen deprivation therapy, glucocorticoid receptor expression and activity increase, compensating for or bypassing AR to restore downstream signaling blocked by drugs.^147^^,^^148^ Williams et al^149^ have reported that SOX2 expression is associated with resistance to NHRSIs. Cells expressing SOX2 demonstrate insensitivity to glucocorticoid receptor signaling inhibition via glucocorticoid receptor regulatory therapy. Mechanistically, the gene encoding glucocorticoid receptor, nuclear receptor subfamily 3 group C member 1 (*NR3C1*), has been identified as a new target of SOX2, which can positively regulate glucocorticoid receptor expression. Moreover, SOX2 affects cancer cell proliferation and survival by regulating cell cycle-related genes *WEE1* and cyclin-dependent kinase 1 (*CDK1*), making PCa cells resistant to NHRSI treatment. Pharmacologically targeting WEE1 (WEE1 inhibitor) combined with androgen receptor pathway inhibitor or glucocorticoid receptor modulators resensitized SOX2-positive PCa cells to NHRSI *in vitro*, and the combination of WEE1 inhibitor and androgen receptor pathway inhibitor substantially retarded tumor growth *in vivo* (Fig. 3B). Schematic diagram of the progression of resistance to endocrine therapy in PCa and its relationship with SOX2 expression is shown in Figure 4.

### Therapeutic potential of targeting SOX2

Experimental results from PCa cells with either overexpression or knockdown of *SOX2* suggest that directly targeting the SOX2 transcription factor could be a promising strategy for inhibiting tumor initiation and progression. However, despite the significant effects observed in cell-based studies using siRNA or shRNA techniques, translating this strategy into clinically effective treatments still presents numerous challenges.

SOX transcription factors recognize DNA with high specificity, forming complexes with partner factors such as paired box 6 (Pax6), Nanog, and Oct4 to bind to and bend the DNA.^150^ Therefore, targeting the DNA-binding capacity of SOX transcription factors or inhibiting their interactions with these partner factors may offer a more effective strategy. Recent studies have shown that certain small molecules, such as Sm4, can specifically inhibit the interaction between SOX18 and its cofactor recombination signal binding protein for immunoglobulin kappa J region (RBPJ), thereby blocking SOX18's transcriptional activity. This discovery not only presents a new approach for anti-angiogenesis therapy, particularly in tumor angiogenesis, but also opens potential clinical applications for therapies targeting SOX18.^151^ Importantly, targeting the protein–protein interactions of SOX proteins, rather than directly targeting DNA binding, offers higher selectivity and reduces side effects. These findings validate the feasibility of transcription factor-targeted therapies and open new avenues for drug development in diseases such as prostate cancer.

In addition to directly targeting SOX2, another potential strategy is to target molecules that regulate SOX2 expression or influence its upstream and downstream functions.^152^ As discussed earlier, we outlined the upstream and downstream molecules of SOX2 and the signaling pathways it mediates. These molecules and pathways regulate SOX2 expression and activate downstream effects that influence tumor cell behaviors, such as proliferation, migration, and invasion. Therefore, identifying and targeting regulatory factors within the SOX2 signaling network—either upstream or downstream—could be a critical strategy. This approach not only inhibits SOX2's tumor-promoting role but also presents new potential targets for cancer therapies.

While these strategies show considerable promise, further research is essential to validate their applicability across various cancer types. Specifically, targeted therapy aimed at SOX2's upstream or downstream regulatory factors must ensure selectivity and safety to avoid disrupting normal physiological processes. Despite the significant therapeutic potential of directly or indirectly targeting SOX2 in cancer treatment, it is important to note that SOX2 also plays critical roles in normal physiological processes. Inhibition of SOX2 could lead to severe side effects, particularly in tissue regeneration and stem cell functions. Inhibiting SOX2 may result in delayed tissue regeneration or dysfunction, underscoring the need for careful targeting. Therefore, identifying targetable factors upstream or downstream of SOX2 and ensuring that these factors can suppress tumor growth without adversely affecting normal tissue are important directions for future research.

## Conclusion and future perspectives

In this review, we briefly describe the role of SOX proteins in PCa, with a particular emphasis on the SOX2 transcription factor. SOX2 plays a crucial role in the initiation and progression of PCa. Compared with benign tumors, SOX2 expression is significantly up-regulated in PCa tissues, correlating with higher tumor grades and poor patient prognosis. SOX2 drives the progression of PCa by promoting cellular proliferation, invasion, metastasis, and resistance to apoptosis. Moreover, SOX2 regulates cancer stem cell characteristics and induces EMT, further enhancing the metastatic potential of PCa cells. These findings underscore the importance of SOX2 as a key molecular participant in PCa and highlight its potential as a therapeutic target.

Targeting SOX2 is crucial due to its regulatory role in PCa metastasis, treatment resistance, and key signaling pathways. As a transcription factor, SOX2 shares the characteristic of being “undruggable”, like many other transcription factors. To date, no molecules have been found that effectively bind to and block the function of SOX2. Therefore, developing strategies to inhibit SOX2 activity in PCa cells by targeting noncoding RNAs or other molecules involved in regulating SOX2 may provide new treatment avenues, particularly for patients with advanced and metastatic disease. Future studies should focus on i) further investigating the specific regulatory mechanisms of SOX2 in PCa and other cancers, particularly its role in the tumor microenvironment; ii) exploring the non-stem cell functions of SOX2 in PCa, rather than merely considering it a stem cell marker; iii) examining the role of SOX2 as an epigenetic reprogramming factor in lineage plasticity; iv) elucidating the interactions between SOX2 and other signaling pathways to identify new therapeutic targets; and v) developing more selective and efficient SOX2 inhibitors, particularly small-molecule drugs targeting its protein–protein interactions.

## CRediT authorship contribution statement

**Guotu Du:** Writing – review & editing, Writing – original draft. **Xiang Huang:** Writing – original draft. **Peng Su:** Data curation. **Ying Yang:** Conceptualization. **Shicheng Chen:** Writing – original draft. **Tianyu Huang:** Writing – original draft. **Neng Zhang:** Writing – review & editing, Supervision.

## Funding

This study was supported by National Natural Science Foundation of China (No. 81860524) and Zunyi Municipal Bureau of Industry, Science and Technology [Zun shi ke he HZ zi (2024) No. 216].

## Conflict of interests

None.
